# Peripheral compound odontoma erupting in the gingiva

**DOI:** 10.1186/1746-160X-9-15

**Published:** 2013-06-11

**Authors:** João AC Hanemann, Denise T Oliveira, Natália Galvão Garcia, Mariana RG Santos, Alessandro AC Pereira

**Affiliations:** 1 Department of Clinic and Surgery, Area Stomatology, Alfenas Federal University, Minas Gerais, Alfenas, Brazil; 2 Department of Stomatology, Area of Pathology, Bauru School of Dentistry, University of São Paulo, Al. Dr. Octávio Pinheiro Brisolla, 9-75, Bauru, SP 170120-901, Brazil

**Keywords:** Peripheral odontoma, Compound odontoma, Erupted

## Abstract

**Background:**

Peripheral odontoma arising in the extraosseous soft tissues is rare and if not removed early, may enlarge over time and eventually erupt in the oral cavity.

**Case presentation:**

A 15-year-old girl presented with “denticles on the gingiva”. During the intraoral examination, seven small tooth-like structures were found. These were exposed in the anterior left gingiva between the permanent maxillary lateral incisor and canine teeth, and the left first premolar was absent. Radiographic examination revealed irregular tooth-like structures without evidence of bone involvement.

**Conclusion:**

The lesion was surgically removed, and the specimens were analyzed histopathologically. The diagnosis of compound odontoma was established.

**Clinical significance:**

This is the twelfth reported case of peripheral odontoma in the gingiva and the first one that erupted in the oral cavity.

## Background

Odontomas are the most common type of odontogenic tumors and they are often associated with permanent or temporary tooth eruption disturbances [1]. According to the latest classification of the World Health Organization (WHO, 2005), two types of odontomas can be recognized: complex odontomas and compound odontomas [2].

Clinically, odontomas are either intraosseous or extraosseous [3-5]. The intraosseous odontomas occur inside the bone and may eventually erupt into the oral cavity (erupted odontoma). Peripheral odontoma arising in the extraosseous soft tissues is rare and have a tendency to exfoliate [5].

We report a case of a 15-year-old girl with a peripheral compound odontoma characterized by multiple rudimentary teeth which erupted spontaneously in the anterior maxillary region. Based in the related English literature, this is the first reported case of peripheral compound odontoma with several teeth erupted in the gingiva.

## Case presentation

**Figure 1:**
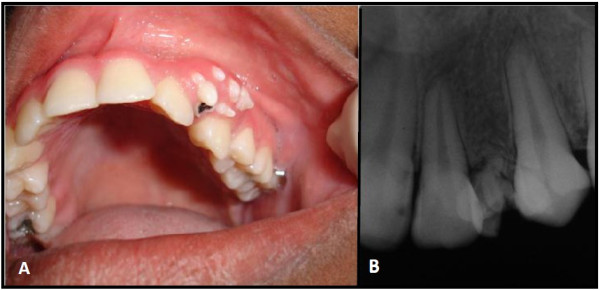
Clinical and radiographic of small tooth-like structures erupted in the gingiva.

**Figure 2:**
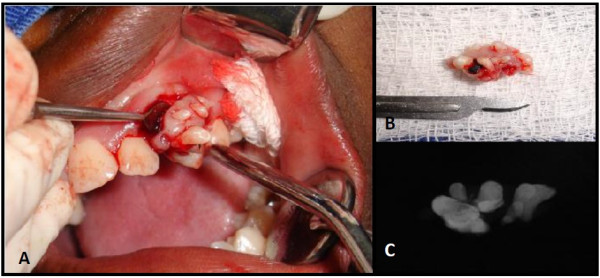
Surgical removal of the lesion.

**Figure 3:**
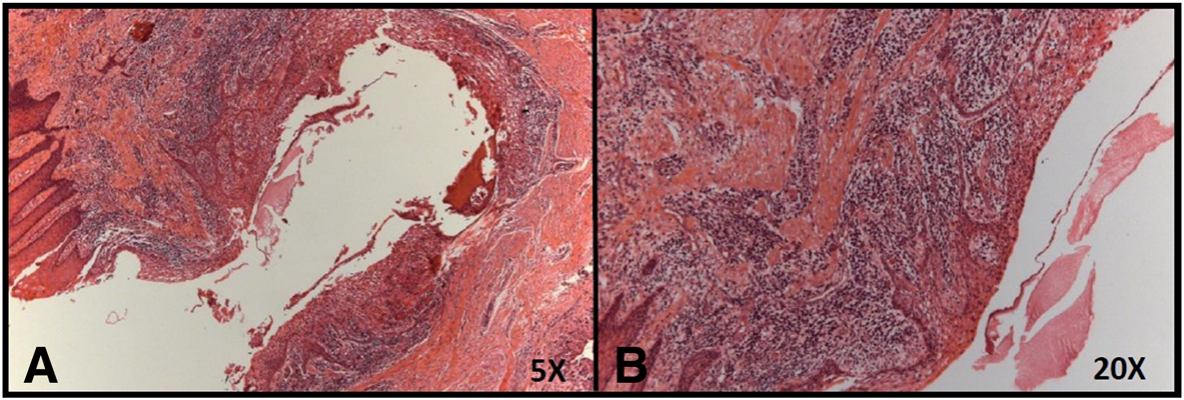
Histopathological analysis of gingival tissue around the small tooth-like structures.

The patient, a 15-year-old girl was referred to the Stomatology Clinic of Alfenas Federal University with the complaint of “denticles on the gingiva”. The patient reported that when she was 12 years old, she perceived the emergence of small asymptomatic mass in the anterior maxillary region and one year later multiple denticles erupted at the injury site. During the intraoral examination, the residual root of a deciduous tooth that had been destroyed by caries lesions and six small tooth-like structures were found exposed in the anterior left gingiva between the permanent maxillary lateral incisor and canine teeth (Figure 1A). The denticles had dental biofilm and the gum around them was shown to be swollen and reddened. Radiographic examination revealed irregular tooth structures composed of crown, root and pulp without evidence of bone involvement (Figure 1B). A clinical diagnosis of peripheral compound odontoma erupting in the gingiva was made. The absence of the left first premolar was also observed. The compound odontoma was surgically removed, and no bony erosion beneath the tumor was detected (Figure 2A-C). The specimens were sent for histopathological analysis which confirmed that it was a compound odontoma with enamel, dentin, pulp chamber, and cementum in the same order of arrangement as that of a normal tooth, surrounded by thin epithelium (Figure 3A). These structures were embedded in dense fibrous connective tissue containing a mononuclear inflammatory infiltrate and bacterial colonies (Figure 3B).

**Figure 4:**
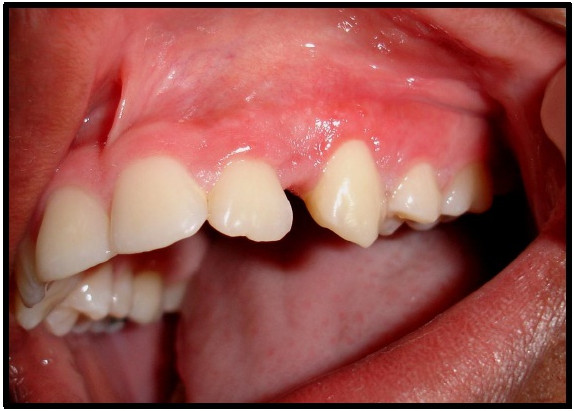
After one month.

Postoperatively there was no complication and the suture was removed ten days after surgery. At this time, there was satisfactory healing of the operated region. After one month, the patient showed complete healing of the lesion and the gingival contour in the region of the lateral incisor and canine teeth had been fully restored (Figure 4). After this she was referred to the dental clinic for completion of her dental treatment. So far, she has shown no signs of recurrence.

## Discussion

Odontomas are generally asymptomatic lesions and they constitute causal findings in the course of routine radiological studies, particularly in the second and third decades of life [6]. Compound odontomas are more frequent than the complex type and they show a predilection for the anterior maxillary region [2,3].

Peripheral or soft tissue odontomas are extremely uncommon with about twelve compound odontomas reported in the related English literature, including the present case, as summarized in Table 1. In this table it may be observed that there is a predilection for children and young persons and in over 50% of cases the lesions affected the palatal region [6-9]. The histogenesis of this type of odontoma has been associated with soft tissue remnants of the odontogenic epithelium such as gingival rests of Serres, which seem to retain the ability to pursue epithelial-mesenchymal interactions that could lead to odontoma formation [6,9]. In our patient, the clinical oral examination showed the residual root of a deciduous tooth that had been destroyed by caries lesions, six small tooth-like structures exposed in the oral cavity and absence of the left first premolar. Thus, it is suggested that the odontoma may have arisen from this premolar germ.

**Table 1:** Reported cases of peripheral developing compound odontoma

**Author/Year**	**Gender/Age**	**Localization**	**Erupted in oral cavity**
**Present case 2013**	F/15 year old	Anterior maxillary region	Yes
**Friedrich et al. 2010**[10]	M/3 year old	Palatal region	No
**Silva et al. 2009**[9]	Case 1- M/8 months	Case 1 – palatal region	No
	Case 2- M/5 months	Case 2 – palatal region	No
**Ide et al. 2008**[8]	F/7 year old	Anterior mandible region	No
**Bernardes et al. 2008**[6]	M/12 year old	Anterior maxillary region	No
**Kintarak et al. 2006**[11]	F/13 year old	Palatal region	No
**Takeda et al. 2005**[12]	F/2 year old	Palatal region	No
**Ide et al. 2000**[7]	M/39 year old	Anterior maxillary region	No
**Ledesma-Montes et al. 1996**[13]	F/3 year old	Posterior mandible region	No
**Giunta et al. 1990**[14]	Case 1- F/5 year old	Case 1 – palatal region	No
	Case 2- M/21 year old	Case 2- posterior mandible	No
**TOTAL**		12 cases	

Although peripheral odontomas have a limited potential for growth, Ide; Shimoyama; Horie, (2000), the case here reported reinforces the idea that if the lesion had not been surgically removed in early developmental stage, it might have erupted into the oral cavity. The eruptive mechanism of peripheral odontoma remains uncertain and it appears to be different from tooth eruption because of the absence of periodontal attachment in odontomas [3].

The majority of erupted odontomas described are intraosseous and related to non-erupted teeth [2-5]. Therefore, it may be postulated that the eruptive force of non-erupted teeth plays an important role in odontoma eruption. In the absence of non-erupted teeth, some authors believe that odontoma eruption is due to severe bone resorption at the site [4]. Others report that the mechanism behind the eruption may involve both the bony remodeling of the jaws and the increase in tumor size over time, since the force required to move the odontoma is not linked to the contractility of fibroblasts, as is the case with teeth [3].

On the other hand, the exposure of denticles from the peripheral compound odontoma in the gingiva, as illustrated in the present case, could represent a tendency to exfoliation, as suggested by Shekar et al., due to its growth over time, rather than a real process of tooth eruption.

Histopathological analysis showed that the tooth-like structures were composed of enamel, dentin, pulp chamber, and cementum in the same order of arrangement as that in a normal tooth. These structures were surrounded by thin epithelium and embedded in dense fibrous connective tissue, demonstrating their peripheral origin. The rudimentary denticles that had erupted from the odontoma had a fragile insertion into the gingival tissue which was clinically confirmed by accentuated tooth mobility. The fibrous connective tissue contained mononuclear inflammatory infiltrate and bacterial colonies was clinically characterized by inflammation of the gums due to the presence of dental biofilm, probably caused by the difficulty of cleaning in the region.

## Conclusion

In summary, due to the rare occurrence of peripheral odontomas, the present case is the first report of an erupted peripheral odontoma. However, it is necessary for dentists to be aware that peripheral odontomas, if not removed early, may enlarge over time and eventually erupt in the oral cavity, compromising both periodontal health and esthetics. Moreover, it is important to note that the increased tooth mobility caused by these lesions may result in the spontaneous exfoliation of these denticles, which may lead to the patient swallowing them.

## Clinical significance

This is the twelfth reported case of peripheral odontoma in the gingiva and the first one that erupted in the oral cavity.

## Consent

Written informed consent was obtained from the patient for publication of this Case report and any accompanying images. A copy of the written consent is available for review by the Editor-in-Chief of this journal.

## Competing interests

The authors declare that they have no competing interests.

## Authors’ contributions

JACH operated and monitored the patient. DTO performed the microscopic diagnosis and assisted in drafting of paper. NGG accompanied the clinical case and drafted the article. MRGS accompanied the clinical case and drafted the article. AACP assisted in the surgery. All authors read and approved the final manuscript.
